# Review article: outcomes in endoscopic sinus surgery

**DOI:** 10.1186/s12901-016-0030-8

**Published:** 2016-08-05

**Authors:** Edward Noon, Claire Hopkins

**Affiliations:** 1 Northwick Park Hospital, Watford Road, Harrow, Middlesex HA1 3UJ UK; 2 Guy’s and St Thomas’ Hospital, Great Maze Pond, London, SE1 9RT UK

**Keywords:** Outcomes, Outcome measures, PROMs, Endoscopic sinus surgery, Chronic rhinosinusitis, Asthma

## Abstract

Chronic rhinosinusitis has a significant impact on health-related and generic quality-of-life, has a high cost burden to both society and patients, and may be associated with absenteeism, loss of productivity and poor respiratory function. Though there is a paucity of level 1 evidence, endoscopic sinus surgery may be considered in medically refractory patients and a variety of objective and subjective outcome measures exist to assess the effectiveness of intervention. We outline the outcome measurements available and review in-depth the published outcomes to date. Furthermore we discuss the literature that indicates that endoscopic sinus surgery can have a positive effect on respiratory function in asthma. How patient selection, timing and extent of surgery, and post-operative care interventions may optimise surgical outcomes is explored.

## Background

Chronic rhinosinusitis (CRS) affects approximately 11 % of people in the UK [1], and may exist with or without nasal polyps. It is defined by the European Position Paper on Rhinosinusitis and Nasal Polyps [2] as inflammation of the nose and paranasal sinuses, characterised by two or more of the following symptoms, persisting for more than 12 weeks:

- blockage/congestion; discharge (anterior or post-nasal drip); facial pain/pressure; reduction in smell;
- and either endoscopic signs of polyps; mucopurulent discharge from the middle meatus; oedema/mucosal obstruction primarily in the middle meatus;
- and/or mucosal changes within the osteomeatal complex and/or sinuses on CT.

CRS has been shown to have a significant impact on quality of life, greater in some respects than other chronic diseases such as angina or COPD [3]. In addition, there are significant direct and indirect costs to both patients and society; a recent systematic review estimated the overall annual economic burden of CRS to be $22 billion USD (direct and indirect costs) in the US [4]. Direct healthcare costs were estimated to lie between $6.9 to $9.9 billion 2014 USD per year and indirect costs as $13 billion 2014 USD per year, with additional annual medication costs borne by each patient prior to surgery ranging between $1,547 and $2,700 2014 USD.

Medical therapy forms the mainstay of management in CRS, but when this fails to improve symptoms or in the presence of actual or impending complications, surgery is usually considered. Endoscopic sinus surgery (ESS) is now considered standard practice, with open approaches rarely considered in uncomplicated disease.

In order to assess the quality of surgical intervention, a variety of objective and subjective outcome measures exist to facilitate this practice and in recent years there has been a growing volume of published literature on outcomes in sinus surgery, particularly from UK and US centres. This review aims to discuss which outcome measures might be considered in the evaluation of endoscopic sinus surgery for CRS and what the published outcomes of surgery are to date. How ESS for CRS may positively influence the disease pattern in patients with asthma, and may decrease the incidence of new diagnoses of asthma will also be discussed. Finally, we consider how perioperative decision-making may alter surgical outcomes, with particular focus on patient selection, timing and extent of surgery, and the choice of post-operative care strategies.

## Outcome measures and the published outcomes of ESS

When considering the outcomes of sinus surgery, it is important to define how these should be measured. ‘Objective’ measures of surgery, such as endoscopic appearances, ostial patency or changes in CT scans, were often reported as the primary outcome in early studies of endoscopic sinus surgery. However, since the evolution of subjective outcome measurements using validated, disease-specific instruments (Patient Reported Outcome Measures – PROMs), there has been a growing acceptance that the patient’s view of outcomes is the most important. In a recent study of both patients’ and physicians’ views on outcome measurement in CRS, 80 % of respondents considered symptomatic improvement as most important [5].

This section aims to identify the important subjective and objective outcome measures, and describe the published outcomes of ESS to date, as determined by each outcome measure.

## Method

A literature search was performed using PubMed with the search terms “Outcomes *AND* (Endoscopic sinus surgery *OR* ESS *OR* FESS *OR* sinus surgery)”. Searches were constrained to English language and adult patients only. Studies that included concurrent rhinoplasty were excluded. The Cochrane ENT online library (ent.cochrane.org) was also searched and relevant studies were considered for review. The European Position Paper on Rhinosinusitis and Nasal Polyps 2012 [2] was searched carefully for any remaining studies not identified by the initial search methods. To be included in this review, studies were preferentially chosen for their higher level of evidence, size of study, clearly defined outcome measures, and those with the outcome measures we describe as their primary outcome.

### Subjective measures

#### PROMs

Patient-reported symptom severity can be most simply recorded using visual analogue scales. Patients are asked to indicate their answer to the question “How troublesome are your symptoms of rhinosinusitis?” and mark their answer at one point on a 10 cm line. Individual symptom severity may also be rated in the same manner.

There are a growing number of disease-specific instruments, e.g. 31-item Rhinosinusitis Outcome Measure (RSOM-31), Rhinosinusitis Disability Index (RSDI), Chronic Sinusitis Survey (CSS), 20-item Sino-Nasal Outcome Test (SNOT-20), or SNOT-22 (which includes nasal blockage and anosmia), which ask patients to assess quantitatively the severity of a number of symptoms. They may form a useful clinical record and allow responses to treatment to be easily monitored; they may facilitate preference-based healthcare and inform whether a patient may be a suitable candidate for surgery. Though the choice of PROM will depend on the clinical setting, a recent systematic review has considered currently available PROMs in CRS and rated the SNOT-22 as the most suitable tool [6]. The general population may experience some of the symptoms included in the instruments; the normal SNOT-22 score has been found to lie between 7–9 [7]. Whilst changes in symptom scores may be statistically significant, whether this translates into clinical significance is defined by the Minimum Clinically Important Difference, the smallest detectable change in symptoms, which equates to a 8.9 point change in the SNOT-22 [8].

A 2006 Cochrane review, updated in 2009, found no evidence to demonstrate superiority of surgery versus continued medical treatment in terms of symptomatic improvement [9].

Only three level 1 studies were included in the review; one compared middle meatal antrostomy with inferior meatal antrostomy alone, while another considered antral washout versus FESS for isolated maxillary sinus disease. Only one study, Ragab et al., compared medical versus surgical treatment in CRS [10]. Ninety patients with symptoms of CRS for 8 weeks were randomised to either FESS and medical treatment, or medical treatment alone. Despite improvements in both groups, there were no significant differences found between the medical and surgical groups in the patient-reported outcomes in their Visual Analogue Scales, SNOT-20 and SF-36 (Short Form-36: see generic health-related QOL section below). However, patients were submitted to surgery having received only 6 weeks of intranasal corticosteroids and alkaline nasal douche and therefore the results are not generalisable to those offered surgery in current practice – i.e. who have failed maximum medical therapy.

Due to the challenges of recruiting to RCTs in surgery, particularly when medical therapy has failed, there is a paucity of Level 1 evidence. However, there are a number of large prospective cohort studies that demonstrate significant benefit from surgery.

In the largest study of its kind, the UK National Sinonasal audit was a multi-centre prospective cohort study from 87 hospitals and 298 UK Consultant Otorhinolaryngologists [11]. Patients aged 16 years or older that underwent primary or revision surgery for CRS with or without nasal polyposis over a six-month period were included. Health-related quality of life was assessed using the SNOT-22 pre-operatively, then at 3, 12 and 36 months’ follow-up. 70 % of the 3128 patients had nasal polyps and of these 52 % had previous sinonasal surgery, compared with 34 % in those without nasal polyps. The mean overall baseline SNOT-22 score in 2852 (91 %) that completed a questionnaire was 42.0, 41.0 in CRSwNP and 44.2 in CRSsNP. At 3 months following surgery, this mean total score reduced to 25.5, a reduction of 16.5, which represents a large improvement in health-related quality of life. Scores for polyp patients were consistently better versus those without polyps at all follow up periods. At 36 months the overall mean SNOT-22 score was 27.7. Of 1459/2797 (52 %) who were available for further follow-up at 60 months, the mean SNOT-22 score was 28.2 [12]. This long-term data clearly illustrates the potential for a lasting effect on health-related quality of life.

Smith et al. [13] reported on a multi-institutional prospective cohort study in patients who had failed 3 weeks of medical therapy, and who elected either continued medical therapy (*n* = 33) or ESS (*n* = 65). A significantly greater improvement was found in the surgical group at 12-month follow up using the RSDI and CSS as outcome measures.

Similarly, in order to evaluate the benefit gained from endoscopic sinus surgery in CRS compared to continuing medical therapy, Smith et al. [14] enrolled 31 patients who failed 3 months of medical therapy for CRS and were listed for surgery. The time patients spent on the waiting list was used as a self-selecting period for continued medical therapy. SNOT-22 scores were recorded at baseline, at 2 weeks prior to surgery and then at 6 and 12 months post-operatively. After a mean of 7.1 months of continued medical therapy prior to surgery, the mean baseline SNOT-22 score of 57.6 significantly increased to 66.1 by the time of surgery. However, following surgery the mean SNOT-22 score was 16.0, a decrease of 50.1 points.

#### Generic health-related quality-of-life (QOL) symptom scores

Generic scores that do not focus on a particular disease state, e.g. Short Form 36 (SF-36) [15], and the EQ-5D [16] assess a range of general physical and emotional symptoms; they facilitate comparison between different diseases, and allow cost-effectiveness to be compared in terms of Quality-Adjusted Life Years (QALYs). However, they lack the sensitivity to detect changes in CRS symptoms, and are less useful in terms of the clinical record.

Despite this, there are emerging studies that demonstrate that surgery has an impact on more global quality of life.

A group from Oregon, USA, enrolled 232 adult patients with CRS over a 5-year period who underwent primary or revision ESS for CRS and used the Short-Form 6D to evaluate patient-reported quality of life [3]. These data were then converted to health state utility values (HUV), which estimates how the general population considers a particular health state. A score of 0 represents death and 1.0 represents ‘perfect health’, and these values may be compared across different conditions and interventions. A difference in the utility value of 0.03 is considered to be the minimal clinically important difference (MCID) [17]. Their cohort’s mean pre-operative health utility value was 0.65, compared to the mean US population of 0.81. At a mean follow-up of 1.5 years following surgery, of the 168/232 (72 %) who completed the post-operate SF-6D, this increased by 0.087 (0.06–0.12) for those who underwent primary surgery, which the authors commented was a similar improvement to that found following coronary angioplasty; and 0.062 (0.04–0.09) in those who had a revision. At 5-year follow up in the 83 (49 %) that responded, the mean utility value was 0.80, demonstrating a significant improvement from pre-operative scores, and equivalent to the US population mean [18].

Health Utility Values were calculated using the SF-6D in 212 patients electing continued medical therapy or ESS for CRS in a recent North American multi-institutional prospective cohort study. Those who elected medical therapy had better baseline health utility but remained stable through the 6 and 12-month follow-up time points, whereas those who underwent ESS after 3 weeks of failed medical management, showed statistically and clinically significant improvements in health utility [19].

Similarly, a group from Boston prospectively enrolled 242 patients with CRS who underwent primary and revision ESS and calculated the HUV using the Euroqol 5-Dimension assessment (EQ-5D) at baseline, then 3, 12 and 24 months after surgery [20]. Compared to the US normal population HUV of 0.85 calculated with the EQ-5D, their patients’ baseline HUV was 0.81, but increased significantly after surgery at 3 months to 0.89, which was maintained at 24 months.

#### Productivity and absenteeism

It has been recognised that the effects of CRS on physical and mental health may translate into absence from work - absenteeism. Any deleterious effect on concentration due to CRS whilst at work is described as presenteeism [21], and is a self-reported measure of loss in concentration via a questionnaire. The total effect of absenteeism and presenteeism may be described as the Lost Productive Time, and the economic impact this has on society can be estimated using the average daily wage of the national population.

There are only a small number of studies quantifying the substantial indirect costs to the patient and society from absenteeism and lost productive time [21, 22]. Only one evaluates the impact of ESS on these indirect costs; 27 patients with refractory CRS who elected to undergo ESS were enrolled in a study to assess the effect of surgery on productivity costs [23]. The total mean lost productive time due to absenteeism, presenteeism and loss of household productivity (due to time spent caring for CRS symptoms), was 75 days per person per year, equating to an annual productivity cost of $9190 per person. After a mean follow up of 15 months, the lost productive time reduced to 28 days and productivity costs significantly reduced to $3373.

### Objective measures

There are a number of objective measures that may be recorded in patients with CRS.

#### Endoscopic grading systems

Numerous scoring systems exist for the endoscopic evaluation of disease in CRS. Though no consensus exists about what the optimal scoring system is, perhaps the most widely used is that devised by Lund and Kennedy, which includes the assessment of polyps, oedema, discharge, crusting and scarring [24]. It has had a particular role in research studies in patients with polypoid disease. Making the observation that 40 % of this system includes the assessment of post-operative findings (scarring and crusting), Psaltis et al. used a modified Lund-Kennedy score using only polyps, oedema and discharge and found that this gave a high inter-rater and test-retest reliability, and importantly correlated well with the SNOT-22 [25].

Djukic et al. presented a study of 85 patients who had ESS for CRSwNP. At 6 month and 12-month follow-up, Lund-Kennedy endoscopy scores significantly improved to 2.8 and 3.7 respectively, compared to a baseline mean of 8.4 [26].

Thirty-one patients in Canada who had failed 3 months of maximal medical therapy continued their medical treatment whilst on the waiting list for surgery. During a mean of 7 months, Lund-Kennedy endoscopy scores significantly worsened from 6.9 to 7.7 but improved to 2.4 post-operatively [14].

#### Recurrence/revision rate

Measuring recurrence has to be defined by a specific end-point. Data on revision surgery is a simple, objective way of doing so; however it could be argued that the *disease* has recurred before the time of revision surgery but defining this point is perhaps more difficult and is partly dependent on frequency of post-operative review.

In the UK National Sinonasal Audit [11] that reported data collected between 2000–2001, approximately 4 % of 3128 patients with CRS who had surgery required a revision procedure within 1 year, and 11 % within 3 years. Of the 1459 (52.2 %) that responded to a 5-year follow-up, it was revealed that 19 % had had revision surgery up to that time [12]. Prior to enrolment in the study, 51.2 % of CRSwNP patients had had previous sinonasal surgery, compared to 34.4 % of those without polyposis, and 46 % overall.

Subsequently a national, multi-centre study from the UK used self-reported patient questionnaires to gather data about timing and extent of previous surgery from 553 patients with CRSsNP and 651 with CRSwNP, recruited from ENT centres. Overall, 43 % reported previous sinonasal surgery, including 29 % in those without polyps compared to 55 % of those with polyps. The mean duration between sinonasal procedures was 10 years [27].

#### Complications

Any benefits of intervention must be weighed up against the risk of adverse events. Complications are usually classified as major (CSF leak; orbital complications including orbital ecchymosis, diplopia or reduction of visual acuity; significant intra-operative or immediate post-operative haemorrhage) or minor (adhesions, infection, minor bleeding and post-operative pain).

The National Sinonasal Audit reported a total adverse event rate of 6.6 %, most of which was related to minor bleeding. Eleven (0.4 %) of 3128 patients had major complications, of which seven (0.2 %) were orbital complications. Five patients had a peri-orbital haematoma and 2 had peri-orbital emphysema. None had a reduction in visual acuity or extra-ocular movements. Two patients (0.06 %) had a CSF leak, which were addressed intraoperatively and a further two returned to theatre because of major post-operative haemorrhage. After multivariate analysis, there was a statistically significant increase in complication rates with increasing SNOT-22 and Lund-Mackay CT scores, and extent of polyposis [28], demonstrating that important subjective and objective outcome measures may be used as a predictor of post-operative outcome when measuring complication rates. This rate of major complications from the UK (0.4 %) compares with a rate of 1.1 % reported in a meta-analysis from 10 years previously of 4691 patients who underwent ESS in the US [29].

There is little data reporting the risk of complications from medical treatment for CRS. Nonetheless, there is a small but important risk of major complications such as gastric ulceration, osteoporosis and immune suppression with the use of systemic steroids, and there is growing evidence of a risk of cardiac death with clarithromycin use in patients with cardiac anomalies. It is essential that future trials capture risk of adverse events in both medical and surgical treatment arms.

#### Olfactory tests

Numerous tests of olfactory function exist, such as the University of Pennsylvania Smell Identification Test (UPSIT) [30], which involve the identification of commonly known odours. Sniffin’ Sticks [31] also permits odor discrimination and olfactory threshold testing. Tests should be able to discriminate between those with normal olfaction, and those with varying degrees of olfactory dysfunction. Their use is more common in research studies than in routine clinical practice.

In a prospective study of the effect of primary ESS for CRS on olfactory function determined by Sniffin’ Sticks testing, Minwengen stratified patients according to mild or more advanced sinus disease based on a Lund-Mackay score of ≤7 and ≥8 [32]. Thirty-eight patients (50 %) had post-operative olfactory assessment out of an initial 76. Though there was a significant improvement in patients with more advanced disease, and no improvement with mild disease, those in the mild group had pre-operative olfactory scores that were nearly normal.

Litvack et al. reported a multi-centre, prospective cohort study of olfactory function using the UPSIT at baseline, and at 6 and 12 months following ESS in 111 patients [33]. In anosmic patients (UPSIT scores 6-18/40), there was a large and significant improvement in olfactory function from a mean score of 9.7 to 21.3 at 6 months, sustained at 12-month follow-up. There was no statistically significant improvement in the hyposmic group and the normosmic patients remained stable, though 26 % of anosmic patients had an increase in UPSIT score of 4 or more. The presence of nasal polyps was a strong predictor of improvement, especially in the anosmic patients.

However, comparing 280 patients who elected continued medical therapy or ESS after failed initial medical management, measurement of Brief Smell Identification Test (B-SIT) scores showed equivalent improvement in both groups [34]. This study data has limited generalisability in European patients as the decision about further treatment was made after a minimum of only 3 weeks, which is contrary to current European guidelines.

### Heterogeneity of outcome measures

The extent of outcome measures is wide-ranging, making familiarity with interpreting data and comparing results from several studies for meta-analysis more challenging. It is likely that one outcome measure alone is insufficient to be able to assess the efficacy of a given treatment, whether novel or part of routine practice. The difficulties that accompany this heterogeneity are currently being addressed by an international expert team of rhinologists, which form part of the COMET group (Core Outcome Measures in Effectiveness Trials) [35]. They aim to develop a ‘core outcome set’ of outcome measures that should be used as a minimum when evaluating new treatments and routine management.

## What is the impact of ESS on respiratory function?

There is now emerging evidence to suggest that addressing the disease burden of CRS surgically may have a positive impact upon respiratory function in patients with asthma.

### Impact of ESS on the incidence of asthma

The healthcare records of 2833 patients with CRS in the USA who underwent primary ESS were reviewed for the presence of coexisting asthma and associated healthcare visits [36]. Significantly more patients had asthma (45.4 %) in those that waited the longest to surgery (≥5 years) following their asthma diagnosis, compared to those who had surgery within 1 year (20.3 %). Whether this data means that treating medically refractory CRS has a beneficial effect on respiratory function, or the greater incidence of asthma is a manifestation of the underlying disease process of the entire respiratory tract is yet to be established.

This group also sought to establish how the incidence of new asthma diagnoses varied in relation to the time between first diagnosis of CRS and primary ESS. The healthcare records of 1204 patients with no pre-existing history of asthma were analysed, which revealed that there was an almost linear increase in the incidence of asthma diagnoses between 9.4 % in 181 patients who had primary ESS between 1–2 years after diagnosis, and 22.4 % in 536 patients who had surgery between 4–5 years after diagnosis. Following surgery, there were significantly fewer new asthma diagnoses in those who had surgery between 1–2 years, versus the 4–5 years group [37].

### Is there a beneficial effect from ESS on pre-existing asthma?

A systematic review and meta-analysis found 22 studies identifying at least one asthma outcome following sinus surgery [38]. Overall asthma symptoms improved in 76 % of patients, and there were also decreases in frequency of asthma attacks, hospitalisations and emergency department visits. The frequency of oral corticosteroid use decreased by 72 %, and inhaled corticosteroids decreased by 28 %. However, no studies identified reported an improvement in FEV_1_ or PEF. Despite this thorough review, the included studies were not controlled against patients who did not have surgery; there was variation in the severity of asthma and extent of surgery; and, whether this apparent improvement in asthma outcomes is sustained is not known.

In 47 patients in Canada with severe asthma who underwent FESS having failed medical therapy for ESS, the number of visits to the asthma clinic in the immediate 12 months prior to surgery compared to those in the subsequent 12 months decreased by 50 % (6 to 3) [39].

## Effectiveness of revision surgery

Treatment for chronic rhinosinusitis is not yet curative and UK data suggests 43–46 % of patients require revision surgery [11, 27]. Given the heterogeneity of disease severity, extent and timing of surgery, and study design, outcomes data on revision compared to primary surgery is understandably variable. However, there does broadly appear to be consistency in the finding that revision surgery seems to provide symptomatic improvement in selected cases.

Patients undergoing primary surgery were shown to be 2.1 times more likely to improve using the RSDI and 1.8 times more likely with the CSS in a prospective cohort study of 302 patients in whom 61 % were revision cases [40].

In a single-centre prospective cohort study of revision versus primary ESS, 167 patients with similar pre-operative Lund-Mackay, RSDI and CSS scores, following surgery both groups experienced significant and comparable symptomatic improvement and there was no significant difference between the two groups measured by the CSS. The revision group’s post-operative RSDI scores were slightly but significantly worse than the primary group, but had similar change scores [41].

In a series of 125 patients undergoing revision FESS in CRS, patients with and without polyps, who had had an average of 2.4 prior procedures in polyp patients and 1.6 in those without polyposis, a significant reduction in SNOT-20 scores of 23.0 points was observed at 2-year follow-up [42]. There was not a comparative cohort undergoing primary surgery however.

In a number of studies comparing primary with revision surgery there has been variation in the revision cohorts with patients undergoing different numbers of previous procedures. To address this, Clinger et al. reported a multi-institutional prospective cohort study of 552 patients undergoing primary and revision surgery [43]. They used the RSDI and CSS to compare the symptomatic benefit following primary surgery, with the first, second, third, fourth and fifth or more revision procedures. Symptom score improvement was better in those undergoing primary surgery versus the revision groups combined. However, no significant differences were found in post-operative improvement between the revision subgroups.

## How can we influence outcomes?

### Patient selection

A recent study examined the value of the pre-operative SNOT-22 in predicting the post-operative SNOT-22 scores of UK patients in the National Sinonasal Audit [44]. Stratifying 2263 patients according to their SNOT-22 scores into 11 groups (0–10, 11–20, etc.) at 3-month follow-up, there was a greater absolute reduction and percentage reduction of scores with increasing pre-operative severity. However those with higher pre-operative scores remained the most symptomatic after surgery. At least 50 % of patients achieved a minimum clinically important difference [8] of 8.9 points whose pre-operative SNOT-22 was at least 20 and overall 66 % of patients experienced this. A North American multi-institutional cohort study of 327 patients with a mean follow-up of 14 months, revealed similar results, supporting the UK data [45].

This knowledge can be used to facilitate shared decision-making and guide expectations about surgery after failure of medical therapy. Certainly, patients with a SNOT-22 score of less than 20 are less likely to achieve a clinically significant benefit, and careful consideration should be given before proceeding with surgery.

### Timing of surgery

#### Symptomatic benefit from earlier surgical intervention

Hypothesising that untreated CRS is a progressive disease, in the absence of any previous similar studies, Hopkins et al. took data on 1493 patients collected prospectively in the UK Sinonasal Audit to evaluate the symptomatic benefit from early primary surgery versus intervention later in the disease process [46]. Duration of symptoms was recorded and SNOT-22 scores collected at baseline, 3, 12 and 60 months following surgery, with return rates of 80 %, 78 % and 49 % respectively. Patients were divided into an Early cohort - less than 12 months since symptoms began, a Mid cohort - 12–60 months, and a Late cohort - more than 60 months. In the late cohort, the mean baseline score was 40.8, significantly higher than 35.8, found in the early cohort. Post-operatively, mean SNOT-22 scores were significantly lower at all follow up periods in the early cohort compared to the mid or late cohort. At 3 and 12 months, this difference appeared to be explained by lower pre-operative scores in the early cohort because the mean change in SNOT-22 scores was similar, but at 60 months a persistent worsening in symptom scores meant that the benefit gained following surgery initially in the late cohort was not sustained. Similar outcomes were found with asthma patients excluded. Furthermore, a significantly higher proportion of patients reached a Minimal Clinically Important Difference of ≥8.9 points in the early group versus the late group at 12 and 60 months. This study indicates that early surgical intervention after a trial of medical therapy may deliver better symptomatic outcomes that are sustained for as long as five years.

#### Earlier surgical intervention and a reduction in prescription and outpatient visit burden

In a further retrospective analysis of the UK Electronic Health Data, Hopkins et al. aimed to evaluate whether there were any differences between 2534 patients having early versus later surgical intervention, with respect to postoperative healthcare utilisation measured by outpatient consultations, and prescriptions related to CRS [47]. The cohorts were assessed up to 5 years following surgery. The overall 5-year mean was 0.85 visits per patient per year in the Early cohort and 1.06 in the Late cohort (*p* < 0.0001). Furthermore, the number of prescriptions related to CRS per patient per year was significantly higher in the Late cohort compared to the Early cohort (0.54 versus 0.36; *p* < 0.0001). These differences in post-operative healthcare utilisation are consistent with those of the study by Benninger [36]. Current European guidelines (EPOS) advocate surgical treatment for CRS when optimal medical management has made no improvement in symptoms after 12 weeks. The number of patients having surgery as late as 5 years and beyond after a formal diagnosis of CRS is therefore surprising but perhaps are a reflection of attempts at cost saving measures within the NHS, which these two studies by Hopkins et al., and that by Benninger et al., suggest are futile in the long term.

### Extent of surgery

A Cochrane review from 2014 aimed to establish whether any Level I evidence existed to elucidate whether nasal polypectomy with additional sinus surgery conferred any benefit over nasal polypectomy alone in the treatment of CRSwNP [48]. Six studies met some of the inclusion criteria but no studies fulfilled all of them and could therefore not be included in a systematic review.

Though a number of studies suggest some benefit from more extensive surgery, heterogeneity in patient characteristics, disease severity and study design makes obtaining level 1 evidence challenging.

In a prospective, randomised trial of 65 patients with CRS with and without NP, patients underwent either limited ESS (uncinectomy, middle meatal antrostomy and uncapping of the bulla ethmoidalis) or a more extensive approach involving clearance of all sinuses with middle turbinate reduction [49]. At 3 and 6-month follow up, despite improvement in all outcome measures, there was no significant difference in outcomes between the two groups, suggesting that a limited approach might be sufficient. However, 29 % of patients were lost to follow-up at 6 months and 75 % by their long-term follow-up, limiting the value of this small study. Jankowski et al. retrospectively evaluated 76 consecutive patients with CRSwNP that had had either a radical ethmoidectomy (‘nasalisation’) (*n* = 39) or a functional ethmoidectomy (*n* = 37) performed by separate surgeons [50]. Patients were posted a questionnaire and asked to grade their nasal symptoms based on a 10-point visual analogue scale. They were also invited for a follow-up visit to have a postoperative endoscopic assessment. Despite a relatively high loss to follow up (15.6 % and 32.4 % respectively), combined polyposis recurrence and revision surgery rates were significantly lower in the nasalisation group (22.7 % versus 58.3 %) at a mean of 4.7 years after surgery in the nasalisation group and 3.8 years in the functional group. The symptomatic benefit was also greater in the nasalisation group (VAS 8.41 vs. 5.69, *p* = 0.002).

A UK group retrospectively evaluated 149 patients with CRSwNP operated on by a single surgeon who underwent ‘extensive’ ESS during a 5 year period, and aimed to establish whether this approach was associated with a lower rate of revision surgery [51]. This was compared to data collected in the UK Sinonasal Audit. Extensive ESS was defined as nasal polypectomy, middle meatal antrostomy, anterior and posterior ethmoidectomy and exploration of the frontal recess. Their cohort included both primary and revision cases. They reported a revision rate of 4.0 % at 36 months after surgery versus 12.3 % (*p* = 0.006) from the National Audit [11]. While the methodology is prone to bias (hospital records would not detect if revision surgery had been performed in another setting), this suggests that further research is needed to determine the optimal extent of surgery.

In a recent multi-centre prospective study from North America, 311 patients undergoing FESS for CRS after failure of medical therapy were enrolled to investigate the effect on extent of surgery on SNOT-22 scores [52]. Patients underwent either ‘complete’ surgery where all sinuses were opened, or ‘targeted’ surgery - any operation less extensive than the ‘complete’ approach. Extent of surgery was determined by the extent of clinical and radiological disease on CT, and the individual surgeon’s judgment, but surgeons were blinded to pre-operative symptom scores. Of the 147 (47 %) that underwent complete surgery, there was a significantly higher prevalence of asthma, ASA sensitivity, nasal polyposis, and intervention was more likely to be revision surgery. After an average follow up of 13 months, the mean total SNOT-22 score for the complete surgery group was 29.3 vs. 57.4 at baseline (a mean improvement of 28.1), and 27.8 vs. 49.8 at baseline for the targeted group (a mean improvement of 21.9). Despite a greater burden of disease outcome measurements pre-operatively, those patients undergoing complete surgery in this study appeared to receive greater benefit compared to those undergoing a targeted surgical approach. However, the difference in SNOT-22 scores did not reach the minimal clinically important difference [8].

### Post-operative care

The evaluation of any beneficial effect on outcomes from post-operative medication use has perhaps received less attention than the initial medical therapy for CRS. However in 2011, Rudmik et al. performed a systematic review of the impact of early post-operative care interventions on short and long-term quality of life outcomes following FESS. In addition to patient-administered medications, post-operative sinus cavity debridement and drug eluting stents/spacers were included in the review [53].

#### Nasal saline irrigation

Six randomised-controlled trials were identified that evaluated the effect of nasal saline irrigations. A level 1b Taiwanese study of 77 patients revealed a statistically significant improvement in symptoms and endoscopic appearances in those with mild CRS who had nasal saline irrigation and cavity debridement versus those with cavity debridement alone, but no difference in those with moderate-severe CRS [54]. The review postulated the likelihood of greater benefit than harm, and together with the low cost and side effect profile, nasal saline irrigation was recommended as a post-operative strategy [53].

#### Sinus cavity debridement

Post-operative sinus cavity debridement is thought to be a valuable part of post-operative care in some centres. A trial of debridement versus no debridement in 60 patients was shown to have a significantly reduced rate of middle turbinate adhesions at 3 months [55]. Ninety patients who were randomised to debridement three times in the first post-operative week or debridement once on day 7 showed a minimal but statistically significant advantage in symptom scores from frequent debridement [56]. A further randomised study found better early symptom scores in 30 patients who had weekly debridement versus fortnightly for 4 weeks [57]. Rudmik’s group recommended sinus cavity debridement as a post-operative intervention but this may be difficult to justify in state-funded healthcare systems given the associated cost and lack of clear symptomatic benefit [53].

#### Intranasal steroids

A number of well-designed RCTs to assess the effect of post-operative nasal steroid sprays exist. The highest quality was a double-blinded, placebo-controlled, randomised controlled trial of fluticasone propionate commencing 6 weeks after FESS [58], which demonstrated significant symptomatic improvement compared to the placebo group at 1 and 5-year follow-up using a visual analogue scale in which patients were asked “How do you feel overall?” [24]. Topical nasal steroid spray was the only post-operative care strategy to be strongly recommended (grade of evidence = A) in Rudmik’s systematic review [53].

#### Other treatments

Further treatment options reviewed were antibiotics, systemic steroids, topical decongestants and drug-eluting spacers/stents but these were considered to be ‘optional’ in the post-operative setting because of either weak/limited evidence for any benefit or the potential for medication side-effects.

As the authors point out, these recommendations should be considered guidelines, rather than a mandatory post-operative intervention for an individual patient. And despite the evidence presented, the exact timing, dose, and delivery device that should be used to administer medication, and in which patients, remain unclear.

## Conclusion

Outcome measures in sinus disease, in particular CRS, have evolved significantly over the past 20 years, with a trend away from objective measurements as the primary outcome and towards patient-reported outcomes. The range of available tools allow us to evaluate the effectiveness of surgery with respect to health-specific and generic quality-of-life, disease burden, healthcare utilisation and respiratory function. Although there is a paucity of level 1 evidence, these outcomes demonstrate significant, sustained benefits from surgery in medically recalcitrant patients. A randomised controlled trial to evaluate the benefit of surgery over continued medical therapy in patients who have failed an initial course of medical treatment, and defining appropriate indications for surgery and the optimal extent should be the focus of future studies.

## Key points

Outcome measures now more commonly report subjective, symptomatic outcomes

There is a paucity of Level 1 evidence to show to any superior benefit from ESS over continued medical therapy, when initial medical therapy has failed

Large, prospective cohort studies suggests benefit exists

ESS may positively influence disease control in asthma

Earlier surgical intervention in CRS after initial medical therapy has failed, appears to deliver improved symptomatic outcomes

## Abbreviations

ASA, acetylsalicylic acid; B-SIT, brief smell identification test; COMET, core outcome measures in effectiveness trials; COPD, chronic obstructive pulmonary disease; CRS, chronic rhinosinusitis; CRSsNP, chronic rhinosinusitis without nasal polyps; CRSwNP, chronic rhinosinusitis with nasal polyps; CSS, chronic sinusitis survey; CT, computed tomography; EPOS, European position paper on rhinosinusitis and nasal polyps, 2012; ESS, endoscopic sinus surgery; FESS, functional endoscopic sinus surgery; FEV_1_, forced expiratory volume in 1 s; HUV, health utility value; MCID, minimum clinically important difference; PEF, peak expiratory flow rate; PROMs, patient-reported outcome measures; QALY, quality adjusted life year; RCT, randomised controlled trial; RSDI, rhinosinusitis disability index; RSOM-31, 31-item rhinosinusitis outcome measure; SF-36, short form-36; SF-6D, short form-6D; SNOT-20, 20-item sinonasal outcome test; SNOT-22, 22-item sinonasal outcome test; UPSIT, University of Pennsylvania Smell Identification Test; VAS, visual analogue scale.
